# Gait Analysis after Total Knee Arthroplasty Assisted by 3D-Printed Personalized Guide

**DOI:** 10.1155/2020/6485178

**Published:** 2020-06-30

**Authors:** Maolin Sun, Ying Zhang, Yang Peng, Dejie Fu, Huaquan Fan, Rui He

**Affiliations:** Center for Joint Surgery, Southwest Hospital, Third Military Medical University (Army Medical University), Chongqing 400038, China

## Abstract

**Background:**

With the development of three-dimensional printing (3DP) technology, the patient-specific instrumentation (PSI) has been widely applied in total knee arthroplasty (TKA). The purpose of this study was to compare the gait parameters of patients with 3DP personalized guide-assisted and standard TKA.

**Methods:**

Retrospective analysis of the advanced knee OA cases in our hospital between June 2017 and June 2018 was conducted. 30 cases received 3DP personalized guide-assisted TKA (group A), and 60 patients who underwent standard TKA during the same period were in group B and group C according to the computed tomography (CT) measurement results, each with 30 cases. Hip–knee–ankle angle (HKA), patella transverse axis-femoral transepicondylar axis angle (PFA), and gait parameters were statistically analyzed. The function was assessed by Hospital for Specific Surgery (HSS) and Knee Society Score (KSS).

**Results:**

The mean follow-up period was 12.3 months in the three groups. The knee max flexion angle at the swing phase of group A was larger than group B and group C (*P* < 0.05), there was no statistically significant difference in other gait parameters. The mean PFA of group A was smaller than that of group B and group C (*P* < 0.05). While, the HKA, HSS, and KSS scores of three groups showed no significant difference.

**Conclusion:**

3DP personalized guide technology could improve the gait parameters after surgery, specifically reflected in the knee max flexion angle at the swing phase. It could also assist in the reconstruction of more accurate patellar tracking and rotational alignment in TKA, avoiding the occurrence of insufficient or excessive extorsion.

## 1. Introduction

Osteoarthritis (OA) is one of the most common diseases leading to disability in the elderly [1]. OA mainly occurs in load-bearing joints, such as the hip, knee, and ankle, and the incidence of OA in knee joint was the highest [2, 3]. The medial compartment of the knee is most easily involved, because the lower limb alignment in normal people presents mild varus, resulting in more load on the medial compartment. OA was mainly manifested as the degeneration and wear of cartilage and subchondral bone. The occurrence of OA is multifactorial, which is related to gender, age, body mass index (BMI), mechanical axis of the lower limb, biomechanical factors, and others [4–8]. OA of the knee should be treated with step care. Drug therapy, high tibial osteotomy (HTO), unicompartmental knee arthroplasty (UKA), and total knee arthroplasty (TKA) can be selected for treatment as the disease progresses.

TKA is the most effective means for treating end-stage OA of the knee [9]. The purpose of TKA is to reduce pain symptoms, correct deformities, restore knee joint motor function, and improve patients' quality of life. A “forgotten knee” is the common goal of patients undergoing TKA and surgeons. In fact, many patients do not obtain a natural knee joint, which leads to much dissatisfaction [10]. As far as we know, patients undergoing TKA are mainly characterized by the loss of range of motion (ROM) and fine motor of the knee compared with healthy people. Therefore, 20% of the patients felt that the TKA did not meet their expectations, and more than half of them had one or more postoperative complications [11].

With the development of three-dimensional printing (3DP) technology, the patient-specific instrumentation (PSI) has been widely applied in orthopedic surgery [12]. In this study, a personalized guide was presented by computer-aided design (CAD) and 3DP technology to assist the accurate surgical transepicondylar axis (sTEA) in TKA. Gait analysis can objectively reflect the functional status of the knee joint better evaluating the efficacy of the surgery and the recovery of patients. Accordingly, the benefits of the guide were evaluated by comparing with the standard TKA in the gait parameters, including kinematic, kinetic, and spatiotemporal parameters, hoping to provide reference for further promotion of this technology.

## 2. Material and Methods

In this study, we performed a retrospective controlled study of the end-stage knee OA cases in our hospital in recent years. The inclusive criteria were (1) advanced knee OA without osteophytes on femoral condyle, (2) OA Kellgren-Lawrence classification grade IV, (3) the structure of the medial and lateral epicondyle of the femur was clearly visible on computed tomography (CT), (4) varus deformity no more than 15 degrees and flexion contracture no more than 10 degrees, and (5) patients without extra-articular deformity of the knee. The exclusion criteria were (1) varus deformity more than 15 degrees and flexion contracture more than 10 degrees, (2) infection of the knee joint, (3) distal femur with huge bone defects, (4) knee valgus deformity, (5) severe extra-articular deformity, (6) with the history of knee surgery, and (7) with periarticular soft tissue dysfunction and neuropathy.

**Figure 1:**
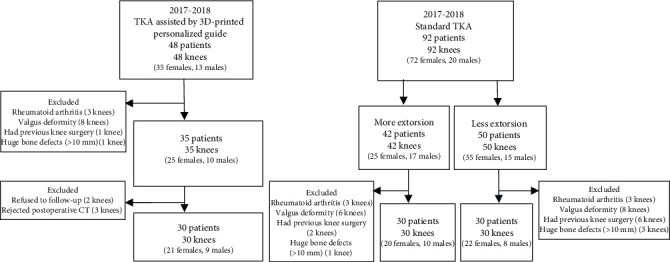
The flow diagram of participants according to the inclusion and exclusion criteria. TKA: total knee arthroplasty.

**Figure 2:**
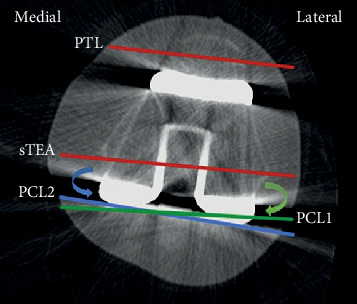
The method to distinguish the insufficient or excessive extorsion of femoral component was measured by CT. Red lines showed the patella transverse line (PTL) and the surgical transepicondylar axis (sTEA), respectively, the green and blue lines represented posterior condyle lines (PCL) under different external rotation states. If it was PCL1 (green line) in the internal rotation position of sTEA (green curved arrow), the patient was assigned to group C; if it was PCL2 (blue line) in the external rotation position of sTEA (blue curved arrow), the patient was assigned to group B.

The end-stage knee OA cases in our hospital between June 2017 and June 2018 were conducted. 48 cases received TKA assisted by 3DP personalized guide. According to the inclusion criteria, 30 patients (30 knees) were finally selected (group A). From 92 cases receiving standard TKA, 60 cases (60 knees) were selected and divided into excessive extorsion (group B) and insufficient extorsion (group C) by measuring the CT, each with 30 cases (30 knees). The flow diagram of participants selected to receive two different types of TKA was shown in Figure 1. The method to distinguish the insufficient or excessive extorsion of femoral component was shown in Figure 2. The characteristics of patients were shown in Table 1.

All surgical procedures were performed by the same senior surgeon. All patients received a LEGION posterior stabilized prosthesis (Smith & Nephew, Memphis, USA); X-ray examination of the full-length lower limb and the thin layer CT of the knee were performed before surgery.

### 2.1. Preparation of 3DP Personalized Guide

**Figure 3:**
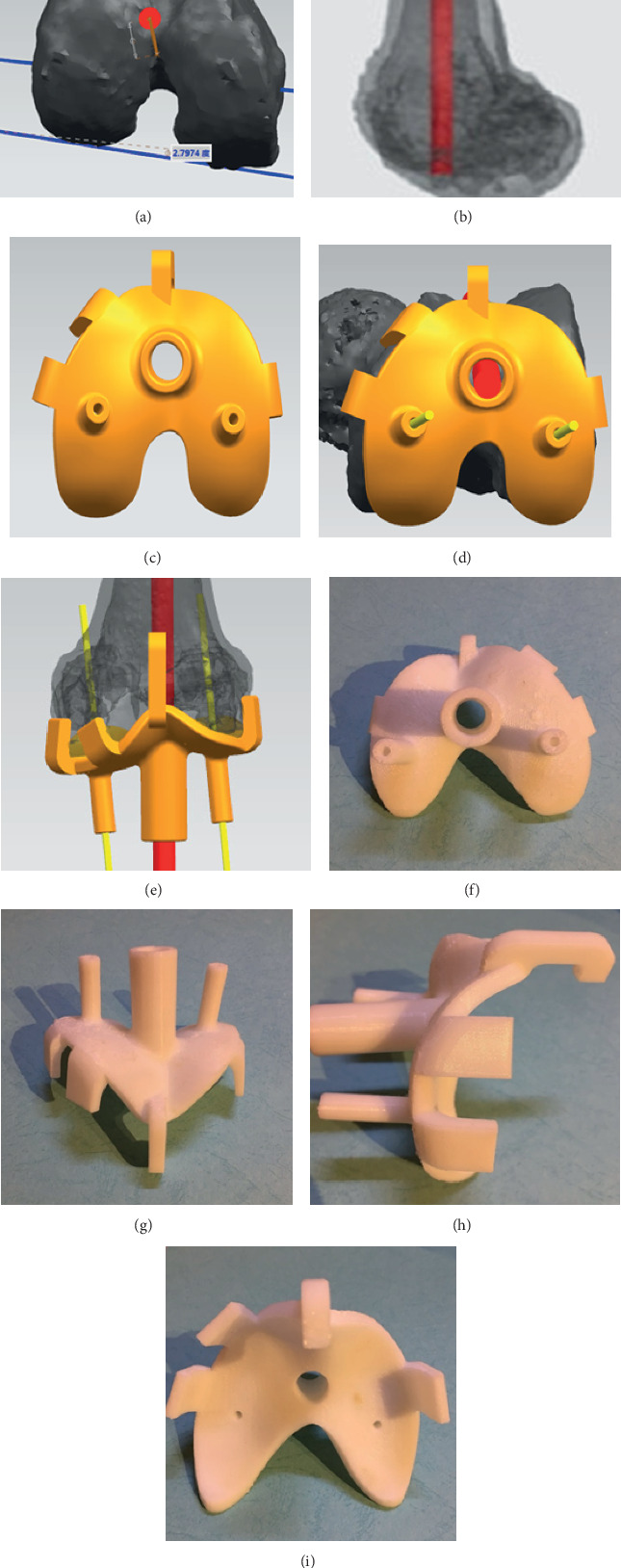
The design and morphology of the guide. (a, b) Determining the surgical transepicondylar axis (sTEA) and the intramedullary positioning. (c–e) The design of the guide. (f–i) The morphology of the guide.

The thin-layer CT scans of the knee were performed before surgery. Digital Imaging and Communications in Medicine (DICOM) data was imported into the Mimics 19.0 (Materialise Ltd., Belgium) to reconstruct the outline of the knee. We synthesized the sulcus area near the medial epicondyle into a circle, and the center of the circle was defined as the lowest point. In the same way, we could confirm the highest point of the prominence on lateral epicondyle. The sTEA was determined by connecting the line of the two points. Then, the data were fed into SIEMENS NX 12.0 (Siemens PLM Software Ltd., Germany) to design the guide. Two points in sTEA were selected as the positioning. The intramedullary positioning was the projection of the femoral anatomical axis (Figures 3(a) and 3(b)). Increasing the depth of the positioning, four fixators around the guide were used to contact with the distal femur firmly (Figures 3(c)–3(e)). Finally, using the laser printer called UP BOX (Tiertime Ltd., China) to print the guide with the biosafe polylactic acid (Figures 3(f)–3(i)).

### 2.2. Key to the Surgical Techniques

**Figure 4:**
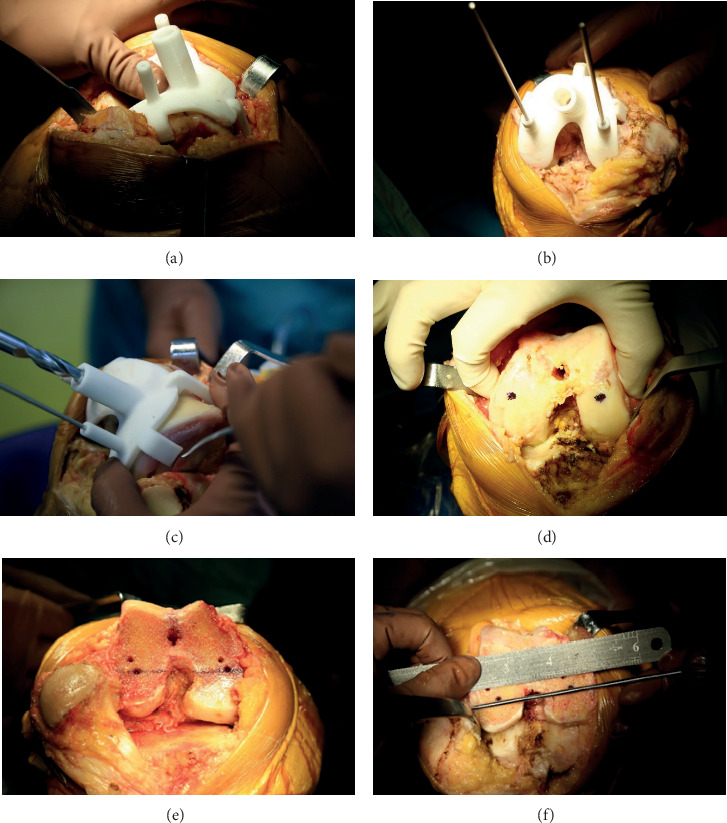
Surgical technique in group A. (a) Placing the personalized guide. (b) Fixing the guide with Kirschner wire. (c) Drilling into the distal femur. (d) Palpating the epicondyles as the first check for accuracy. (e) Completing the distal femoral cuts. (f) Observing the relationship between the epicondylar axis and sTEA as the second check for accuracy.

In group A, the personalized guide was first placed on the distal femur surface (Figure 4(a)). The guide was fixed with the Kirschner wire and sTEA was marked (Figure 4(b)). Then, drilling into the distal femur for intramedullary guide was performed (Figure 4(c)). As the first check for accuracy, the epicondyles were palpated and the sTEA confirmed by the guide was observed (Figure 4(d)). The distal femur cut first and then the tibial cut were completed (Figure 4(e)). Next, as the second check for accuracy, the relationship between the epicondylar axis determined by the femoral rotation guide and the sTEA confirmed by the personalized guide (Figure 4(f)) was observed. For TKA in group B and group C, all participants received standard 3 degree external rotation bone resection for posterior condyles to accomplish the surgical procedure. In all cases, no patella replacement was performed.

### 2.3. Gait Analysis

**Figure 5:**
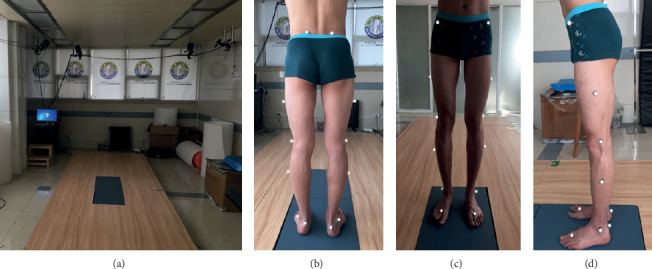
Gait analysis equipment and the position of reflective markers. (a) Gait analysis equipment. (b–d) The position of reflective markers.

The Vicon motion analysis system with 8 cameras (Vicon, Oxford Metrics Ltd., UK) was used to capture kinematic and spatiotemporal parameters of patients [13]. In addition, a 10-meter walkway embedded with two force platforms (AMTI, Watertown, MA, and Kistler Ltd., Switzerland) was used to collect the kinetic parameters [14, 15]. The camera was adjusted to 150 Hz and the force platform to 3000 Hz to collect the data of gait pattern [14, 15] (Figure 5(a)).

All cases were measured for standard anthropometric dimensions including height, weight, the knee width, and the ankle width. 16 passive reflective markers were affixed to the bony landmarks of the patient's pelvis and lower limbs with double-sided adhesive tape which was based on the conventional gait analysis protocol of Kadaba et al. [15] and Phinyomark et al. [16]. According to the Plug-in Gait (PiG) model (Figures 5(b)–5(d)), 4 reflective markers were located at the spina iliaca; 6 markers at the bilateral lateral femoral epicondyles, the midpoint of the cruses, and thighs; and 6 markers at the bilateral ankles, heels, and forefoot regions [17]. The subject was asked to stand on the force platform; a static calibration trial was collected. Then, we start to build dynamic models and capture gait analysis data including kinematic, kinetic, and spatiotemporal parameters. Finally, at least 6 sets of test data meeting the requirements were selected for gait analysis.

### 2.4. Postoperative Treatment

All patients underwent straight leg raise, ankle pumps, and walking as early as possible after surgery. CT and X-ray examination of the full-length lower limb were reexamined one month after the operation. Hospital for Specific Surgery (HSS), Knee Society Score (KSS) scores, and gait analysis were performed one year after surgery.

### 2.5. Assessment of Results

Outcomes of gait analysis, clinic, and radiology were statistically analyzed. The quantitative data was described by using the mean, and one-way analysis of variance (ANOVA) was used. When significant differences in ANOVA were identified, the least-significant difference (LSD) tests were applied to see if there was any difference between any two groups (SPSS, version 20.0; IBM, Chicago, IL, USA). Significance level was set as *P* < 0.05.

## 3. Results

In this study, all patients in three groups were followed. The mean follow-up period was 12.3 months (range 12–14 months). There was no significant difference between the three groups in age, sex, height, weight, and BMI. The absolute value of the patella transverse line-femoral transepicondylar axis angle (PFA) in group A was smaller than that of the other two groups (*P* < 0.05). However, there was no statistically significant difference in PFA between group B and group C. No significant intergroup differences in hip–knee–ankle angle (HKA), HSS, and KSS scores (function) were observed among the three groups (Table 2).

At the final follow-up, the knee max flexion angle at the swing phase of group A was greater than that of the other two groups (*P* < 0.05); there was no statistically significant difference in other kinematic parameters among the three groups. In terms of kinetic and spatiotemporal parameters, there was no significant difference among the three groups (Table 3).

No prosthetic loosening, incision, and infection complication occurred in the three groups.

## 4. Discussion

### 4.1. Determine the More Accurate sTEA

The instability of the prosthesis due to the wrong rotation axis of the knee is an important cause of TKA failure [18]. Many clinical studies have confirmed the rotation axis fluctuates near sTEA [19]. Many doctors mainly palpated the ends of the medial and lateral collateral ligament to determine sTEA. However, the results of sTEA can fluctuate in the 10° range [20]. Besides, some doctors suggested to refer to the 3° external rotation of the posterior condyle axis to simulate sTEA [20], which was the method adopted by group B and group C. But the included angle between the posterior condyle axis and sTEA would fluctuate between -3° and +8°. Others believed that the white side line was perpendicular to sTEA [21]. In addition, the advent of electromagnetic navigation could prevent human error, while it increased the risk of bleeding and infection [22].

This personalized guide mainly solved the problem of inaccurate femoral rotation alignment in standard TKA. It adopted the technique of preoperative navigation, built a CAD model through the CT data, and made PSI through 3DP technology to assist in obtaining precise sTEA. The 3D femur model reconstructed by a computer was observed from multiple views, and sTEA was marked by the lowest point of medial epicondyle and the highest point of lateral epicondyle. After that, sTEA was shifted in a spatial coordinate system and intersected with the section of distal femur. Finally, sTEA was designed on the guide to realize the visualization, and the goal of personalized design was thus achieved.

### 4.2. Improvement of Gait Parameters

The knee max flexion angle at the swing phase of group A was larger than that of group B and group C. This was because the improvement of femoral rotation alignment assisted by the personalized guide was conducive to better patellar tracking and increased patellofemoral joint contact area. As in this study, the PFA in group A was significantly smaller than that of the other two groups, which was closer to 0°. Berger et al. found that the patellar tracking could be quantitatively evaluated by knee ROM [23]. The better the patellar tracking, the longer the distance the patella slides in the trochlear and the larger the ROM. Under the premise of proper prosthesis and correct surgical technique, postoperative active ROM of the knee might be affected by scar tissue, rehabilitation exercise, psychological status, and other factors. The passive ROM would interfere with the actual ROM result due to the external force and subjective influence of the tester [24]. By the method of gait analysis, the motion pattern of the knee in multiple gait cycles were collected from different perspectives by cameras placed in different positions. The model constructed by reflective markers simulates the walking of the patient, and the results of the gait analysis could reflect the actual gait pattern of the slight changes of knee movement through computer computation [25].

Except for the knee max flexion angle at the swing phase, no difference was found in other gait parameters. This might be related to the insufficient sample size and experimental design. When collecting gait data in the laboratory, due to the limited space, the walking condition of patients in daily life could not be fully displayed. Meanwhile, the shorter walking distance of platform might not fully reflect the objective gait pattern of patients. In fact, no matter how well patients recover after TKA, their results of gait analysis would not exceed that of healthy people. Compared with the healthy controls in the study of Blakeney et al., all cases in our study showed slower walking speed, shorter stride length, and lower cadence. This might be related to postoperative pain, three-point crutch gait, and patient avoidance in pursuit of better test results [26].

In order to obtain more objective results, the tester should stress to subjects that they should not deliberately step on the force platforms but should walk according to his daily gait patterns [27]. At the same time, patients should walk straight from the beginning of the walkway to the end of it and should not stop halfway until receiving the “stop” signal from the tester. In addition, according to previous experience, data within 5% of the average value of the subjects' gait parameters should be selected for the study, and the extreme value should be taken out, so as to make the conclusion more objective [27]. The first step of patients on the force platforms must be a complete step in order to reduce the incidence of data loss [28]. Of course, the researchers could adjust the beginning of the walkway according to the subjects' actual length of lower limbs, height, and walking speed, so that their feet touched the force platform as much as possible. To obtain complete data, we asked the subjects to put their hands over their shoulders during the test to avoid arm swings that would block the markers.

### 4.3. Satisfactory Clinical Efficacy

Varus deformity was corrected, and the lower limb alignment returned to normal in group A. There was no significant difference in the KSS and HSS scores among the three groups. The results showed that insufficient or excessive extorsion of the femoral component within an acceptable range would not affect the overall short-term clinical efficacy.

### 4.4. Limitations

Firstly, previous studies have confirmed that the rotation axis fluctuates dynamically around sTEA during the knee movement [29]. In this study, a single sTEA was used as the reference standard, which might lead to inaccuracy in partial angle range. Secondly, the effect of gender on gait parameters was not considered in this study. McClelland et al. had shown that gait parameters of females with TKA were closer to normal than males [30]. Moreover, the effect of BMI on gait parameters was not discussed. Mulhall et al. and Jarvenpaa et al. discovered that obese patients had poorer mobility and less ROM after surgery compared with nonobese patients [31, 32].

## Figures and Tables

**Table 1:** Characteristics of patients.

	Group A	Group B	Group C
Total (*n*)	30	30	30
Sex			
Male(*n*)	9	10	8
Female(*n*)	21	20	22
Mean age (yr)	68.7 (55-76)	67.5 (57-77)	67.7 (54-80)
BMI (kg/m^2^)	26.7 ± 2.1	26.4 ± 2.0	26.5 ± 2.3
Mean HKA (°)	172.4	171.3	172.6
Mean mMPTA (°)	88.2	87.7	88.0

BMI: body mass index; HKA: hip–knee–ankle angle; mMPTA: mechanical medial proximal tibial angle.

**Table 2:** Radiological and clinical results of three groups at the final follow-up.

	Group A	Group B	Group C	*P* value (group A vs. group B)	*P* value (group A vs. group C)	*P* value (group B vs. group C)
HKA (°)	179.2 ± 3.0	178.9 ± 2.2	178.1 ± 2.6	n.s.	n.s.	n.s.
PFA (°)	0.5 ± 0.3	2.1 ± 0.9	−1.8 ± 0.7	0.000	0.000	n.s.
HSS score	94.4 ± 5.7	93.8 ± 6.2	94.8 ± 5.5	n.s.	n.s.	n.s.
KSS score (function)	92.7 ± 9.6	93.8 ± 10.2	91.5 ± 9.8	n.s.	n.s.	n.s.

HKA: hip–knee–ankle angle; PFA: patella transverse axis-femoral transepicondylar axis angle; HSS: Hospital for Special Surgery, KSS Knee Society Score; “−” indicates that the angle opens inwards.

**Table 3:** Gait parameter results of the three groups at the final follow-up.

	Group A	Group B	Group C	*P* value (group A vs. group B)	*P* value (group A vs. group C)	*P* value (group B vs. group C)
Kinematic parameters						
Knee max flexion angle (swing, °)	59.7 ± 6.0	51.7 ± 5.1	52.2 ± 5.3	0.000	0.000	n.s.
Knee mean abduction-adduction angle (swing, °)	2.0 ± 5.1	1.7 ± 5.7	2.4 ± 4.6	n.s.	n.s.	n.s.
Knee mean internal-external angle (stance, °)	1.1 ± 2.9	0.8 ± 3.2	1.1 ± 3.1	n.s.	n.s.	n.s.
Spatiotemporal parameters						
Walking speed (m/s)	77.0 ± 22.6	78.7 ± 23.1	76.6 ± 21.4	n.s.	n.s.	n.s.
Stride length (cm)	85.6 ± 16.8	84.7 ± 16.1	86.1 ± 17.2	n.s.	n.s.	n.s.
Cadence (steps/min)	106.05 ± 16.9	107.2 ± 15.1	108.7 ± 14.5	n.s.	n.s.	n.s.
Kinetic parameters						
Knee rotation moment (N m/kg)	0.01 ± 0.03	0.01 ± 0.01	0.01 ± 0.02	n.s.	n.s.	n.s.
Knee varus moment (N m/kg)	0.05 ± 0.06	0.05 ± 0.07	0.05 ± 0.07	n.s.	n.s.	n.s.
Knee flexion moment (N m/kg)	0.17 ± 0.10	0.19 ± 0.09	0.18 ± 0.11	n.s.	n.s.	n.s.

## Data Availability

The data used to support the findings of this study are available from the corresponding author upon request.
